# Very low-dose vemurafenib maintenance for cardiac Erdheim Chester disease

**DOI:** 10.1007/s44313-025-00075-5

**Published:** 2025-04-11

**Authors:** Abhijeet Kumar Agrawal, Pronamee Borah, P. D. Rath, Rahul Naithani

**Affiliations:** 1 Department of Medicine, Datta Meghe Institute of Higher Education & Research, Wardha, Maharashtra India; 2 Department of Hematology and Bone Marrow Transplant, Paras Health, Gurugram, Bharat India; 3 Department. of Rheumatology, Max Hospital, Saket, New Delhi, India

**Keywords:** Vemurafenib, Erdheim Chester Disease

Erdheim Chester disease (ECD) is a rare form of non-Langerhans cell histiocytosis, with approximately 1,500 cases reported as of 2022 [1]. These numbers are expected to increase with increased awareness of ECD, which is characterized by tissue infiltration by foamy histiocytes, leading to fibrosis [2]. Cardiac involvement is observed in approximately 37% of cases and can include valve abnormalities, rhythm or conduction defects, and periaortic fibrosis along the entire course of the vessel [3]. The most common finding is infiltration of the right side of the heart (including pseudotumors) in half of the patients [4]. Although asymptomatic patients can be monitored, symptomatic patients and those with organ involvement require treatment. Vemurafenib is a potent inhibitor of the kinase domain of mutant *BRAF*. Vemurafenib has shown activity in ECD with activating mutations in *BRAF* [5, 6]. Treatment with vemurafenib 960 mg twice daily is advised to be continued until disease progression or the development of unacceptable toxicity [7]. This imposes a substantial economic burden on patients. Here, we report a sustained response to a significantly lower dose of vemurafenib in our patient.

A 67-year-old female with known diabetes mellitus and hypothyroidism presented with off and on palpitations and swelling in the bilateral lower limbs for 1 year (left > right), associated with erythema. The patient was diagnosed with atrial flutter. A 2D echocardiography revealed soft tissue density along the pericardium. A positron emission tomography (PET) scan in October 2018 revealed a 10.2 × 7.7 cm mediastinal mass, sclerotic lesions in the bones, and a hypodense lesion in the thyroid. Cardiac magnetic resonance imaging (MRI) in March 2019 revealed a mediastinal mass of 11.3 × 7.9 cm, encasing her right atrium (Fig. 1). The biopsy showed aggregates of foamy histiocytes and was positive for CD68 and CD163, but negative for CD1a, S100, and B-RAF immunohistochemical staining. A somatic *BRAF* missense mutation with a V600E alteration in exon 25, with a mutant allele frequency of 4.9%, was detected along with an *ASXL1* mutation. Fine-needle aspiration cytology of the thyroid lesion was unremarkable. ECD was diagnosed in March 2019. Vemurafenib was initiated at a dose of 240 mg twice daily and was gradually increased to 480 mg twice daily over the next 4 months. Two months later, a repeat PET scan showed a reduction in the size of the mass to 8.5 × 6.2 cm. Hence, the vemurafenib dose was reduced to 240 mg twice daily. Sixteen months after the initiation of therapy, the patient underwent an uneventful hip replacement. Yearly PET-CT and cardiac MRI revealed sustained responses. Two years later, the vemurafenib dose was further reduced to 240 mg/day. Cardiac MRI performed 5 months later revealed a further reduction in the size of the mass when the vemurafenib dose was reduced to 240 mg/day every alternate day. A cardiac pacemaker was implanted approximately 4.5 years after the initiation of therapy. A repeat cardiac MRI performed 1 year later (Fig. 2) revealed only small residual soft tissue thickening along the pericardium and right atrial walls, abutting the superior and inferior vena cava, the coronary sinus with loss of the intervening fat plane, and further subtle mild reduction in soft tissue thickening in the mediastinum. The latest MRI (in 2024) revealed further reduction in soft tissue thickening in the mediastinum (Fig. 3).

**Fig. 1:**
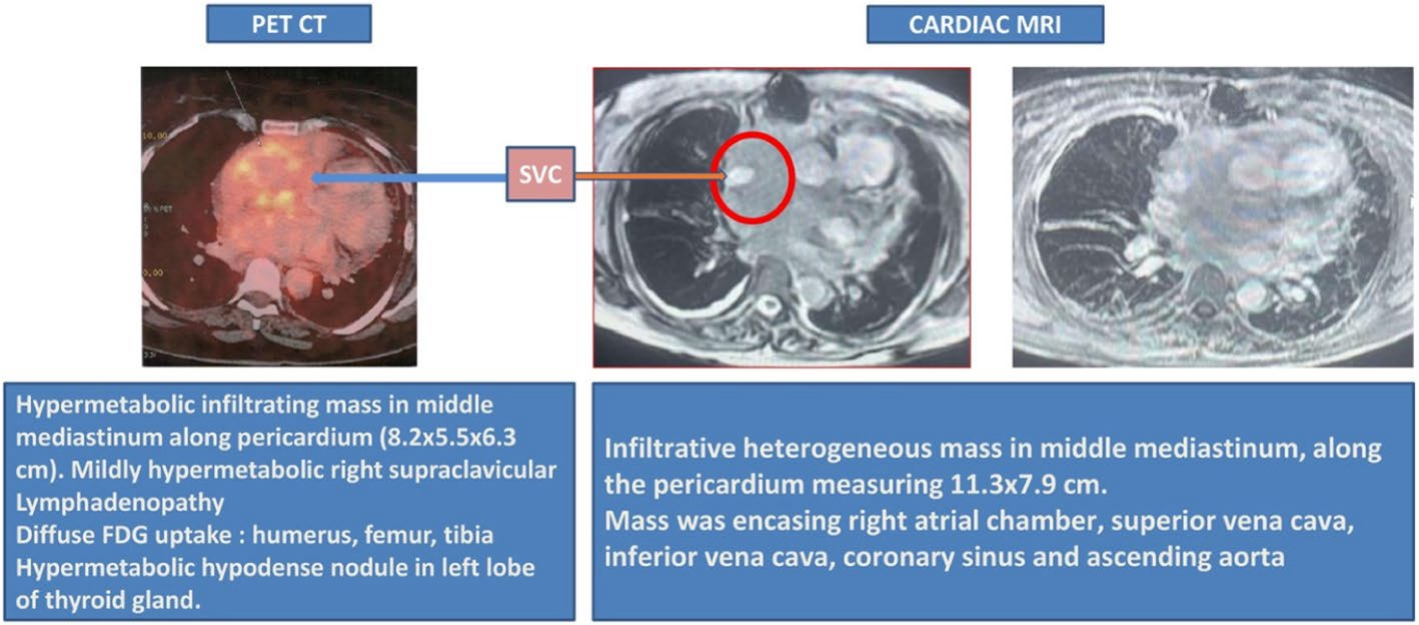
PET-CT and cardiac MRI showing a huge mass in the middle mediastinum along the pericardium at diagnosis. FDG, fluorodeoxyglucose; MRI, magnetic resonance imaging; PET/CT, positron emission tomography/computed tomography

**Fig. 2:**
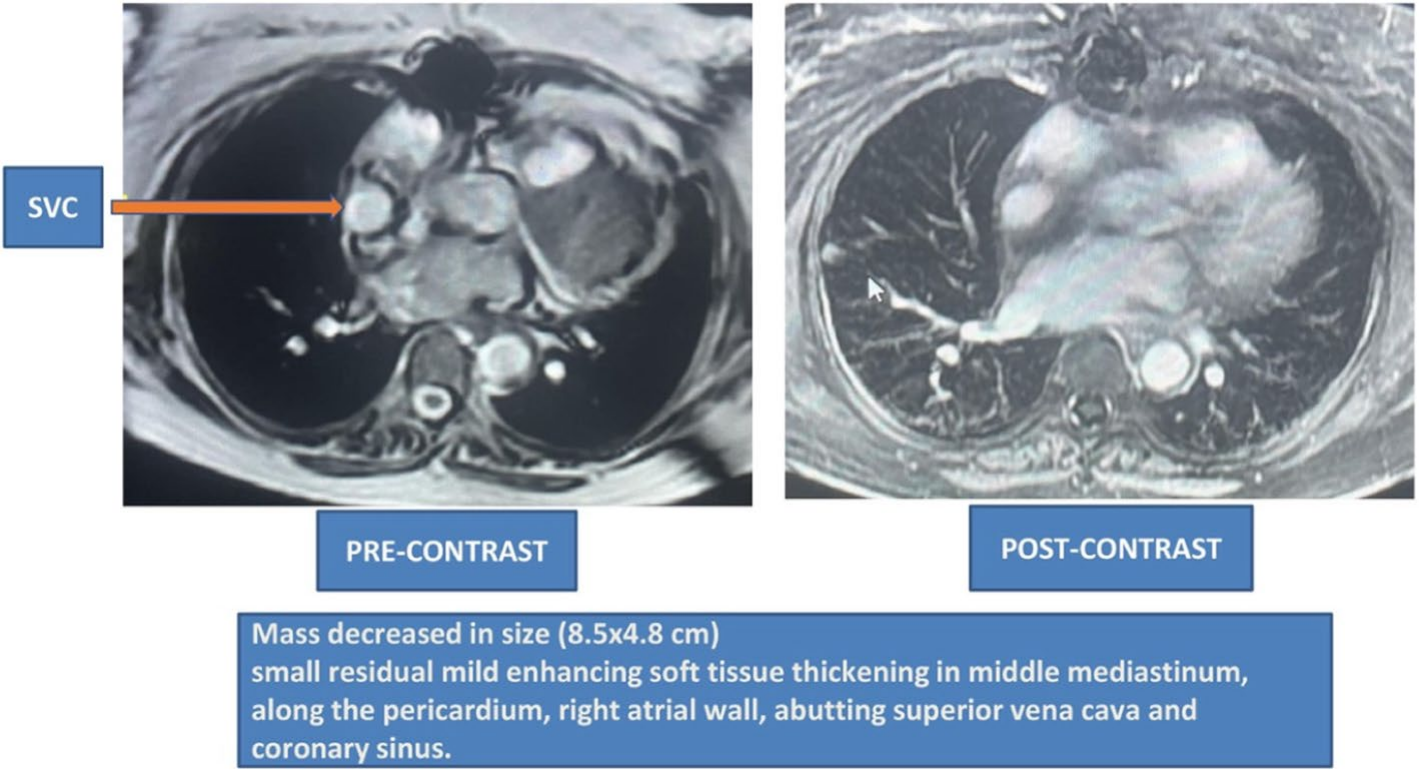
Cardiac magnetic resonance imaging showing a residual mass in the middle mediastinum with no avidity. SVC, superior vena cava

**Fig. 3:**
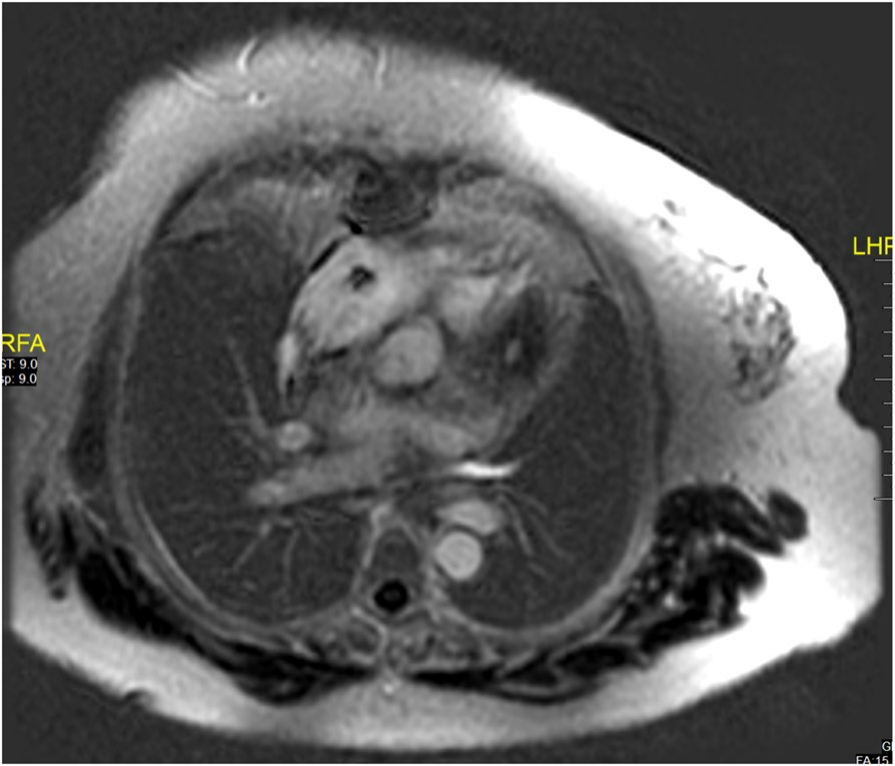
Cardiac magnetic resonance imaging showing subtle residual minimal soft tissue thickening in the middle mediastinum along the pericardium and right atrial walls abutting the SVC with no avidity

Given the persistent response, the vemurafenib dose was further reduced to 240 mg/day twice a week, and the patient remained at this dose for 2 years. During the initial phase of therapy, the patient complained of excessive hair fall, loss of eyebrow hair, and development of multiple warts. Early in the course of treatment, she was diagnosed with flexor tenosynovitis and was started on hydroxychloroquine. None of the adverse effects led to the discontinuation of vemurafenib. At this point, the plan was to continue the dose indefinitely. No new adverse effects were noted during dose reduction. Hair fall continued, but there were no warts at reduced doses.

It is difficult to establish definite clinical management guidelines owing to the rarity of ECD and the small sample size of clinical studies. Cardiac involvement in ECD is usually associated with disseminated disease and insufficient response to therapy, whereas regression of cardiac involvement is associated with improved survival [8]. The discontinuation of vemurafenib is discouraged as most patients have been observed to relapse after treatment interruption. Cohen et al. reported the long-term outcomes of a vemurafenib stoppage study. They reported relapses in 11 of 12 patients with ECD at a median of 5 months (range 1–12 months) [9]. In clinical practice, a lower dose is recommended to circumvent poor compliance due to adverse effects [10, 11].

Ruan et al. described 23 patients with ECD treated with a starting dose of vemurafenib ranging from 960 mg twice daily to 240 mg once daily. Patients on 960 mg twice daily required early dose reduction because of adverse events, and patients on 240 mg once daily had to be titrated to achieve a response [10]. At a median follow-up of 2.5 years, the data suggested that vemurafenib doses that are 50% of the FDA approval may be adequate in achieving a response in patients with ECD. Another series of three patients was described where responses were observed with a half-dose of dabrafenib and a lower dose of cobimetinib [12]. Vemurafenib binds to the ATP-binding site of BRAFV600E and inhibits downstream phosphorylation of ERK. It has a half-life of 57 h [13]. This should allow for less frequent dosing with vemurafenib.

We report the 5.7-year follow-up of this patient. The starting dose was 480 mg twice a day (50% of the recommended dose), and once a response was achieved in 6 months, the dose was further reduced by half. Vemurafenib is expensive and has significant adverse effects at the recommended dosage. The patient maintained remission at 480 mg/week (3.57% of the recommended dose of 13,440 mg/week).

The findings of a single case report cannot be easily generalized to the community. Therefore, generalizing this finding and recommending it as standard practice would be difficult. However, this case provides a window into the possibility of a sustained response in an ECD (non-CNS involvement) case with the least side effects and economic burden when using a significantly lower dose of vemurafenib.

## Data Availability

No datasets were generated or analysed during the current study.
